# The negative association between health status and digital health literacy among Chinese youth: a cross-sectional study on Chinese general social survey

**DOI:** 10.3389/fpubh.2025.1682986

**Published:** 2026-01-08

**Authors:** Peng Hou

**Affiliations:** 1 Department of Physical Education, Xidian University, Xi 'an, China; 2 School of Humanities and Social Science, Xi’an Jiaotong University, Xi’an, China

**Keywords:** health literacy, youth, health, physical exercise, socioeconomic status, heterogeneity

## Abstract

**Background:**

The healthy growth of youth is related to the future of the country. However, under the social pressure, the time and space of Chinese youth are extremely compressed, and there are generally health problems such as obesity and declining physical fitness. Based on the above issues, the study uses data from the China General Social Survey (CGSS 2021) to systematically examine the relationship between contemporary Chinese youth health status and digital health literacy, with the aim of improving the health level of Chinese youth.

**Methods:**

An empirical study was conducted that using from the International Social Survey Project (ISSP) health module of the China General Social Survey (CGSS 2021). The Chinese youth group was selected as the research sample, and used OLS model to analysis the relationships between the health status of contemporary Chinese youth and their digital health literacy.

**Results:**

There is a significant negative correlation between the health status of Chinese youth and their digital health literacy (*p* < 0.01). By replaced the independent variables and used the AIPW method to overcome the sample selection problem, it shows that the empirical results of this study have good robustness (*p* < 0.05). Exploratory analysis further indicates that physical exercise plays a masking role in the relationship between Chinese’s youth health status and their digital health literacy (*p* < 0.01). The impact of health status on digital health literacy is only significant in the youth group with high socioeconomic status (*p* < 0.01).

**Conclusion:**

This reverse relationship reveals new characteristics of Chinese youth’s health behavior in the digital age. That is the lower health status, the higher their digital health literacy, which forms a causal paradox. Heterogeneity analysis shows that digital inequality still exists among Chinese youth. Therefore, it is recommended that youth pay attention to the combination of physical exercise and digital health literacy in daily health management, enhance their ability to obtain, understand, and apply health information, and achieve sustainable health improvement.

## 1 Introduction

As the backbone of national development and rejuvenation, the growth and development of the youth are tied to the future of the country and the nation. With the acceleration development of the digital society, the digital literacy level of Chinese youth group has improved significantly. According to the 55th Statistical Report on the Development of Internet in China, the number of Internet users in China has reached 1.108 billion. Among them, 61.2% possess at least one kind of digital literacy, especially youth (1, 2). At the same time, their health literacy and level are not optimistic. Under the heavy social pressure, the time and personal space of contemporary youth are extremely constrained, and there are generally unhealthy lifestyles such as prolonged sitting, staying up late, and lack of exercise. Meanwhile, it is difficult for the youth to modify these unhealthy habits (3). As a result, phenomena such like “youthfulness of chronic diseases” and “sudden death from overwork” are occur frequently, and health risks continuous increase become a health problem that cannot be ignored (4–6). It is evident that the digital health literacy of Chinese youth has not improved in tandem with their overall digital literacy.

In this context, digital health literacy has gradually become an important topic in health research. Norman proposed the concept of digital health literacy at first, defining it as the ability to search, understand, and evaluate health information from electronic resources and use it to solve health problems, emphasizing its distinction from general digital literacy (7). With the continuous promotion of “Internet plus medicine,” digital health services such as online consultation and health consultation have developed rapidly (8). The 55th Statistical Report on the Development of Internet in China shows that 418 million people in China us Internet-based healthcare services, accounting for 37.7% of all Internet users (9). It can be seen that digital health services have achieved remarkable results in improving health accessibility and inclusiveness after the practice of real digital integration (10, 11). However, facing problems such as the proliferation of health information and insufficient information judgment ability, youth also face cognitive dissonance and behavioral gaps when using digital health tools (12, 13).

Therefore, what is the relationship between youth health status and digital health literacy? There are two controversial views in existing research: one supports the “correlation theory,” which believe that there is a positively correlated relationship between health status, behavior and health literacy (14). The other type of research supports the “irrelevance theory,” which believe that high education, high income, and socioeconomic status determine the level of digital health literacy (15). Although both views have empirical support, there are generally two limitations: one is to ignore the particularity of the youth group and assume that the effects between different groups are consistent. The second is the failure to consider differences in social and economic status within the group, which presupposes homogeneity in individual responses. The Chinese youth group in the digital age exhibits its unique individual characteristics, whether it is “Lie down” or “Competition,” “Inheritance” or “Rely on the older adults,” all of which highlight the unique characteristics of the localization and produce diverse digital behavior patterns (16). Meanwhile, the research has found that socioeconomic status factors such as education level and income are key variables that affect health outcomes and access to social resources (17). Obviously, based on relevant research from other countries, Chinese youth have their own unique social characteristics that cannot be viewed uniformly (14).

To address the research controversies and concerns about the health status of contemporary Chinese youth, this manuscript attempts to start from the perspective of the behavioral subjects of youth health and digital health literacy. Through a nationwide sampling survey data [China General Social Survey (CGSS) 2021], systematically analyzes the relationship between Chinese youth’s health status and digital health literacy, as well as the heterogeneity characteristics within the group, in order to provide theoretical support and empirical evidence for improving the health of Chinese youth in the digital age.

## 2 Theoretical hypothesis

Digital health literacy is a product of the integration of health literacy and digital literacy development. Health literacy refers to the important ability of individuals to obtain, understand, and apply health information to improve their quality of life, and is widely recognized as a significant influence on health levels (18–20). Digital literacy was first proposed by Israeli scholar Eshet Alkalai in 1994. Initially it referred to the ability to utilize digital tools and unlock their potential, with significant implications for individuals and society. Some scholars believe that digital literacy includes the comprehensive ability to acquire, understand and use Internet information, and regard it as an important basis for individuals to adapt to society in the digital era (21, 22). On this basis, Norman first proposed the concept of electronic health literacy, emphasizing the ability to access and use health information within a digital environment (7). With the introduction of the Global Strategy for Digital Health (2020–2024), the concept of digital health literacy has gradually taken shape, emphasizing the comprehensive ability of individuals to acquire, evaluate, and apply health information and technology in the digital environment to maintain health (23, 24). The further refinement of concepts from health literacy to digital health literacy also highlights the diversification of health needs in different eras.

As the “natives” of the digital society, contemporary Chinese youth generally possess certain digital literacy, but their digital health literacy is still relatively weak. Sasha reviewed relevant literature on the relationship between health status and health literacy systematically, concluded that there is a correlation between health status and health literacy (25). The previous research has shown that artificial intelligence algorithms accurately push consumption-inducing and entertainment content through “digital traces,” further compressing the physical activity space and time of youth, causing contemporary youth to gradually become addicted to digital technology, and then ignoring the importance of digital health literacy (26–28). Meanwhile, some scholars believe that digital media has improved the convenience of life, but has given rise to unhealthy lifestyles such as prolonged sitting, staying up late, and lack of exercise, that may lead to risks such like obesity and subhealth. This gradually led some people to pay attention to the cultivation of digital health literacy (29, 30). It can be seen that with the continuous improvement of digital health services, individual health status and development trends will also affect the interaction and diffusion of health information online and offline to a certain extent (31). The relationship between health status and digital health literacy among youth, as well as how to enhance their health management capabilities, still needs further exploration.

In response, two representative theoretical viewpoints have emerged: the “correlation theory” and the “irrelevance theory.” Scholars who hold a positive attitude toward “correlation theory” believe that individuals with relatively good health levels will promote their digital health literacy. Empirical research has found a positive correlation between self-rated physical health and health literacy. As physical health improves, it helps to further enhance individuals’ self-management capabilities (32). Conversely, poor physical health leads to lower health literacy levels. Some studies have also pointed out that health is a prerequisite for the application of electronic health literacy (33). When individuals continuously promote healthy behaviors and actively solve their own health problems, they can actively seek and obtain correct health information that affects their physical condition, thereby improving their electronic health literacy (34). Additionally, healthy older adults are more likely to recognize digital information on the basis of physical health, thereby improving their digital health literacy and increasing their willingness to access health promoting resources online (35). Meanwhile, self-health behaviors and perceived social support can predict individuals’ digital health literacy, promoting further improvement (36). In the view of “correlation theory” scholars, individual differences in health status can have more feedback effects on digital health literacy. On the other hand, some studies tend to lean toward the negative attitude of “irrelevance theory,” believing that health status is not related to digital health literacy. The research believes that a series of objective factors such as higher education, high income, and socioeconomic status affect the level of digital health literacy (15). Meanwhile, some scholars have found that although some older adults are proficient in using digital apps such as WeChat, the majority of them still lack understanding of health products in the digital sector (37). It can be seen that due to the uneven individual health status at present, some people show a reserved or skeptical attitude toward the recognition and improvement of digital health literacy.

Although the above research provides valuable insights, it mainly focuses on the middle-aged and older adults, and has not yet fully explored the behavioral characteristics of the youth group. At the same time, with the increasing trend of digitalization in society, there is no clear empirical conclusion on whether the improvement of health status can promote the digital health literacy level of youth. There is still a lack of systematic analysis on whether there is a cognitive and practical gap between health behavior and information use, and whether digital health literacy truly plays an empowering role. Therefore, it is necessary to focus on the youth group, further clarify the true effectiveness and mechanism of digital health literacy.

There are four hypotheses proposed in existing literature regarding the impact mechanism of youth health and digital health literacy. Firstly, the social support mechanism suggests that clustered interpersonal relationships can provide a demonstration effect in the process of individual health level changes, leading to individual self-efficacy levels and ultimately affecting digital health literacy (38). Secondly, the information acquisition mechanism, based on the diversified diffusion of health information on digital network platforms, enables individuals to access and share health information resources and learning methods, enhancing the accessibility of health information and affecting digital health literacy (39). Thirdly, the mechanism of healthy eating believes that dietary habits and cognitive levels are interrelated, promoting the mastery of health knowledge and influencing health literacy (40). Fourthly, the mechanism of physical exercise believes that scientific physical exercise is closely related to an individual’s physical and mental health level (41). It can be seen that physical exercise is an important factor related to health status and digital health literacy. However, existing literature has paid more attention to the first three types of mechanisms, and empirical evidence has also obtained reliable conclusions. Regarding the mechanism of physical exercise, considering the controversial views in existing research, especially the “existence anxiety” of time poverty and spatial deprivation in the Chinese youth population, there is still a lack of systematic empirical research on its mechanism, which constitutes the space for further exploration in this research.

Overall, it has been found that the academic community has not yet reached a consensus on the relationship between Chinese youth health status and digital health literacy, and there are generally three shortcomings that make it difficult to clearly explain the relationship between the both.

Firstly, the research subjects and groups need to be improved. The current research mainly focuses on the middle-aged and older adults, neglecting the young population who originally have high digital literacy but whose health condition continues to deteriorate. Some studies have found that youth in the digital society may neglect the acquisition of health information due to their “health confidence,” showing a disconnect between cognition and behavior, and lacking the execution and practical ability to improve their health level (28, 42). Meanwhile, considering that alleviating health anxiety has gradually become an important motivation for individual groups to obtain health information. This may be the key to the failure of youth’s digital health literacy to translate into health benefits. Therefore, the manuscript proposes that whether there is a negative correlation between youth health and digital health literacy needs to be empirically explored in subsequent research.

Secondly, there are still shortcomings in the exploration research. Although the previous research has focused on the importance of mechanisms such as information acquisition and social support for youth health, the exploration of “physical exercise” is still insufficient compared to this special group of youth, especially among those with high digital literacy and weak exercise habits. Given the correlation between health status and physical exercise, and considering the “existential anxiety” of time poverty and spatial deprivation among youth, the manuscript considers that physical exercise has a potential suppressing effect between health status and digital health literacy, which deserves further examination.

Thirdly, there is a lack of heterogeneity assessment. Existing research mainly focuses on the overall effect, and has not fully revealed how socioeconomic status such as education level and income, as well as individual differences, affect the effectiveness mechanism of digital health literacy. These social structural variables may profoundly shape the relationship path between youth health and digital health literacy. Therefore, it is necessary to conduct heterogeneity analysis on youth with different individual characteristics and socioeconomic status in empirical research, in order to better clarify how the health status has different influence on digital health literacy of contemporary Chinese youth.

## 3 Methods

### 3.1 Data sources

The empirical data used in this manuscript is from the International Social Survey Project (ISSP) health module of the China General Social Survey (CGSS 2021). The survey was carried out by the China Survey and Data Center of Renmin University of China, aiming to summarize the long-term trend of social change by systematically collecting data from society, communities, families and individuals. CGSS 2021 covers 28 provinces in China, and the data has strong sample representativeness and randomness. The data includes key variables such as digital literacy, physical exercise behavior, socioeconomic status, and personal characteristics of Chinese youth, which is very suitable for the analysis needs of this manuscript. This research is based on the definition of youth age in the “Medium-and Long-Term Youth Development Plan (2016–2025)” issued by the Central Committee of the Party of China and the State Council. This document is an official guidance document issued by the Chinese government, formulated with reference to the characteristics of Chinese youth. Simultaneously considering the age distribution of CGSS 2021 survey subjects. The manuscript extracts the 18–35 age group as the analysis object. Referring to the processing method of previous literature, based on the needs of the research objectives, only retained the youth group aged 18–35. Meanwhile, the cases that answered “unable to select,” “refuse to answer” related to all variables in the manuscript were eliminated and deleted two missing values (43). This resulted in a valid sample of 615 after removing missing values for key variables and performing data cleaning.

### 3.2 Variable measurements

#### 3.2.1 Dependent variables

The dependent variable of this manuscript is digital health literacy. Referring to the measurement index framework of digital literacy in the research of van der Vaart and Drossaert (44). This research believes that when setting the digital health literacy, a single item is not enough to measure the characteristics of digital health literacy. Therefore, use the questionnaire question “Have you frequently searched for information on the following aspects online in the past 12 months. Mainly including: “a. Information about healthy lifestyle”; “b. Information related to anxiety, stress, or similar factors”; “c. Information about vaccination.” The above items attempt to represent the access ability, understanding ability, and online health information application ability in the digital health literacy of the youth group. The total score reflects the overall performance of the measurement dimension and emphasizes overall accumulation (45). Therefore, add up the three items to determine the level measurement values in construct validity. The three items are assigned values of 1–5 from low to high, so the dependent variable ranges from 3 to 15. The higher the score, the higher the level of digital health literacy.

#### 3.2.2 Independent variables

The core variable of this manuscript is health status, which is translated into self-rated health score by referring to the research method of Tanaka and Johnson (46). Since the health status mentioned in this research is not a single physical health, this variable is measured by the question “In general, what do you think of your health status (Health here includes physical and mental health)” in the questionnaire, which reflects the comprehensive evaluation of the respondents’ physical and mental health at the subjective level. The respondents’ answers range from excellent to poor, and are assigned values ranging from 1 to 5. The lower the score, the better the health status.

#### 3.2.3 Mediating variables

This manuscript mainly selects physical exercise as an intermediate variable in exploratory research. Physical exercise is an important variable between health status and digital health literacy. In the questionnaire, “Have you often taken part in physical exercise in your spare time in the past year?” is used as the measuring device, in which the respondent answers that the frequency is increased from less than every day and is assigned a value of 1–5. The higher the score, the more frequent the physical exercise.

#### 3.2.4 Control variables

The control variables mainly include the demographic characteristics of contemporary youth, economic characteristics and provincial characteristics, which are all included in the analysis as dummy variables in the manuscript. Drawing on existing research conclusions, the influencing factors of digital health literacy are usually related to individual characteristics and social characteristics (17, 47–50). Therefore, the demographic characteristic variables controlled in this manuscript include the respondents’ gender, marital status, registered residence and political status. As the analysis object is limited to the youth group, the age distribution is relatively concentrated, so it will not be further divided and explored. In terms of gender, female youth were coded as 0 and male youth as 1. In terms of marital status, unmarried youth were coded as 0 and married youth as 1. In terms of registered residence, the code of youth with agricultural household registration is 0, and the code of youth with non-agricultural household registration is 1. In terms of political status, the youth who are members of the Communist Party of China are coded as 1, and the rest are coded as 0. Economic characteristic variables include education level, work situation and economic situation. In terms of education level, the code for high school and below (including high school) is 0, and the code for higher education than high school is 1. In terms of work situation, the unemployed youth are coded as 0, the working youth are coded as 1. In terms of economic situation, the youth below the average level are coded as 0, and the youth above the average level (including average level) are coded as 1. The provincial characteristic variables are the information of the respondent’s province, which are also included in the model in the form of virtual variables for control. The descriptive statistical analysis is shown in Table 1.

**Table 1:** Variable definitions and descriptive statistics, *n* = 615.

Variable	Variable descriptions	Mean/%	Standard deviation	*n*
Dependent variable				
Digital health literacy	From low to high level 3–15	7.407	2.008	615
Core independent variable				
Health condition	Excellent = 1, Very good = 2, Good = 3, Not good = 4, Poor = 5	2.585	1.011	615
Replaceable independent variable				
Health concerns	Never = 1, rarely = 2, sometimes = 3, often = 4, always = 5	1.584	0.821	615
Control variable				
Gender	Male = 1, Female = 0	44.6%	0.497	615
Marital status	Married = 1, Unmarried = 0	46.8%	0.499	615
Education level	Higher education than high school = 1, High school and below = 0	53.5%	0.499	615
Registered residence	Non-farm household registration = 1, Agricultural household registration = 0	44.7%	0.498	615
Political status	Party member = 1, Others = 0	8.3%	0.276	615
Work situation	Job = 1, No job = 0	60.0%	0.490	615
Economic situation	Average level and above = 1, Below average level = 0	70.1%	0.458	615
Mediating variable				
Physical exercise	Never = 1, Once a month or less = 2, Many times a month = 3, Many times a week = 4, Every day = 5	3.102	1.289	615
Province	Province, dummy variable	–	–	–

### 3.3 Methods and models

This manuscript first analyzes the correlation of Chinese youth health status on digital health literacy, then explores the intrinsic relationship of Chinese youth health status on digital health literacy, and finally clarifies the heterogeneity of Chinese youth health status on digital health literacy to verify the integrity of the research problem. In addition, considering endogeneity issues such as selection bias, the research chooses to establish an empirical strategy through augmented inverse probability weighting (AIPW) propensity score analysis. AIPW can obtain more robust estimates than traditional propensity score matching through weighted propensity score matching analysis in the counterfactual framework, which is beneficial for verifying the explanatory power of empirical results.

#### 3.3.1 OLS regression

Digital health literacy is a continuous variable with a value of 3–15, respectively. The OLS model estimates the parameters in a regression model by reducing the sum of the squared errors between the predicted and observed values. Compared to the logit/probit model, the OLS model does not have large deviation in significance and impact direction. Meanwhile, the regression results of the OLS model can present marginal effects more intuitively (51). Based on this, this manuscript uses OLS model to estimate digital health literacy, and constructs the model as follows:


$$Y_{i}=\beta_{0}+\beta_{1}X_{1i}+\beta_{2}X_{2i}+\mu_{i}$$ (1)

In Equation 1, *Y*_i_ represents youth digital health literacy; *β*_0_, *β*_1_ and *β*_2_ are coefficients to be estimated; *μ*_i_ are random disturbance terms.

#### 3.3.2 Exploratory analysis test: PROCESS

**Figure 1:**
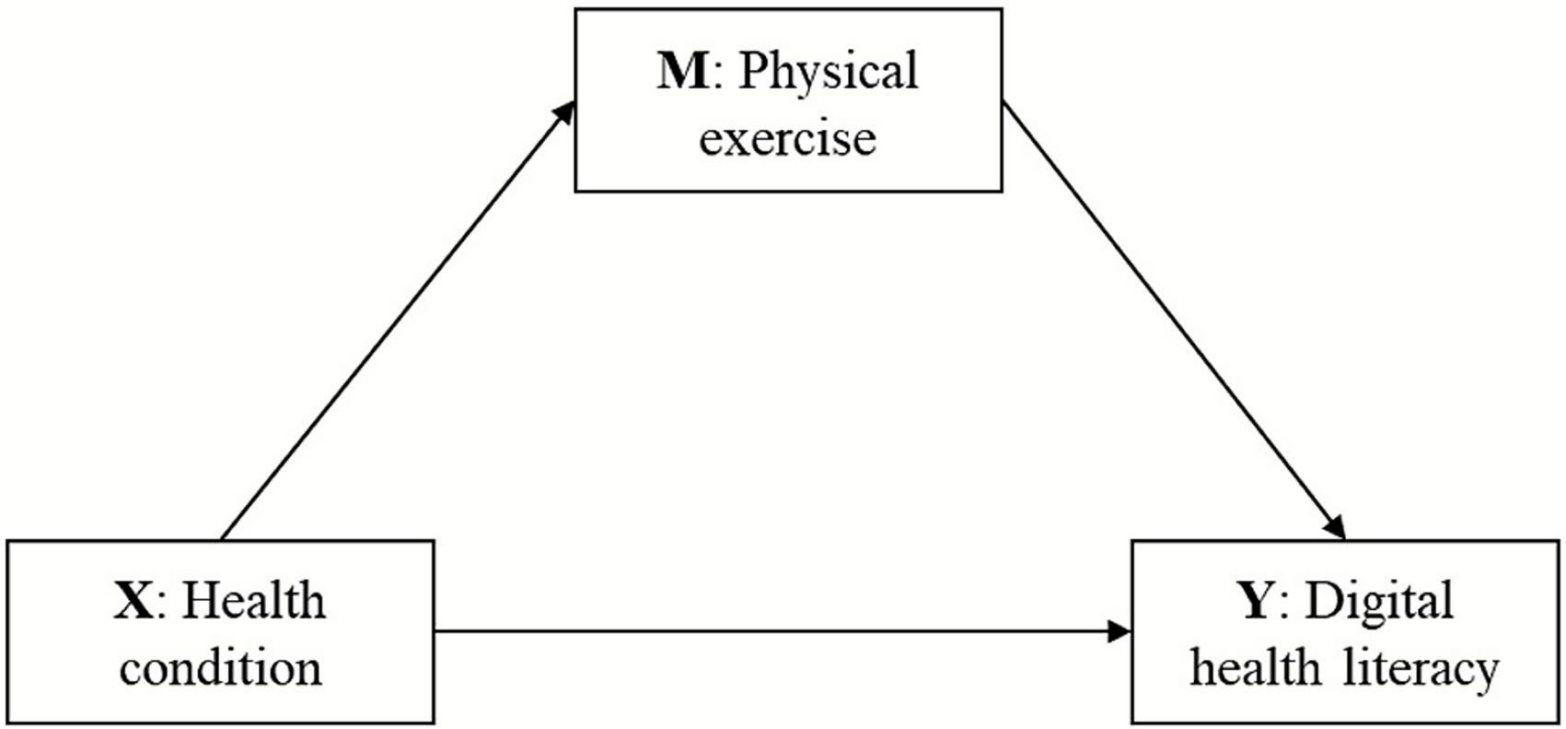
Exploratory analysis roadmap.

It is important to note that causal inferences cannot be drawn from cross-sectional data. So, the current mediation analysis is exploratory. As an exploratory analysis, this manuscript uses Model 4 of the PROCESS v4.2 macro in SPSS to obtain the total effect, direct effect, indirect effect size, and mediation effect ratio of the independent variable on the dependent variable, in order to test the intrinsic relationship between contemporary youth health status and digital health literacy (52, 53). As shown in Figure 1.

## 4 Results

### 4.1 Benchmark regression

The research takes the Chinese youth health status as the independent variable and digital health literacy as the dependent variable. To construct a gradually nested OLS regression model to investigate the relationship between youth health status and digital health literacy. See Table 2 for the estimated coefficients of the regression model. After embedding all control variables, the DW value of the model is 1.975, and the VIF value is 1.033. There are no autocorrelation and multicollinearity problems, indicating that the model is well constructed. At the same time, it can be seen from the regression results in Table 2 that there is a significant negative correlation between the health status of youth and their digital health literacy (*p* < 0.01). That is the worse the health status of youth, the higher their digital health literacy will be. Meanwhile, digital health literacy increases 2.617 units for every unit decrease in health status. Compared with model 1, in model 2 and model 3, the intensity and significance of the relationship between health status and digital health literacy are basically the same.

**Table 2:** Regression results of digital health literacy on Chinese youth health status.

Variable	Digital health literacy		
	Model 1	Model 2	Model 3
Health condition	0.219*** (0.080)	0.192** (0.079)	0.210*** (0.080)
Gender		−0.675***	−0.663***
		(0.164)	(0.169)
Marital status		−0.094	−0.072
		(0.168)	(0.177)
Education level		0.244	0.263
		(0.172)	(0.179)
Registered residence		0.016	−0.008
		(0.168)	(0.171)
Political status			−0.145
			(0.304)
Work situation			−0.005
			(0.173)
Economic situation			0.264
			(0.178)
Constant	6.839***	7.117***	6.886***
	(0.221)	(0.278)	(0.322)
Province	Yes	Yes	Yes
*t*	2.751	2.430	2.617
DW			1.975
*β*	0.110	0.097	0.105
*F*	7.571***	5.560***	3.769***
*R^2^*	0.012	0.044	0.047

^**^p < 0.05, ^***^p < 0.01; Robust standard error in brackets.

In order to ensure the robustness of the results of Chinese youth health status and digital health literacy, the independent variable was replaced to test. The question “How often have health problems affected your work or other daily activities in the past 4 weeks?” is used to measure, which reflects the health problems of the respondents, and also reflects the health status of the respondents from the side. Among them, the respondents’ answers are never always, assigned a value of 1–5. The higher the score, the worse the health status. After embedding all control variables, the model was substituted into the model for regression. The DW value of the model was 1.980, and the VIF value was 1.010. There were no autocorrelation and multicollinearity problems, indicating that the model was well constructed. The estimated coefficients of the regression model are shown in Table 3. It is found that there is still a significant negative correlation between youth health status and digital health literacy (*p* < 0.05). Meanwhile, digital health literacy increases 2.153 units for every unit decrease in health status. It can be seen that the relationship between youth health status and digital health literacy is relatively robust.

**Table 3:** Robustness checks of the association between Chinese youth health status and digital health literacy.

Variable	Digital Health Literacy		
	Model 1	Model 2	Model 3
Health condition	0.225** (0.098)	0.202** (0.097)	0.210** (0.098)
Gender		−0.689***	−0.676***
		(0.164)	(0.170)
Marital status		−0.101	−0.077
		(0.168)	(0.177)
Education level		0.243	0.264
		(0.172)	(0.179)
Registered residence		0.028	0.011
		(0.168)	(0.171)
Political status			−0.152
			(0.305)
Work situation			−0.017
			(0.174)
Economic situation			0.219
			(0.177)
Constant	7.050*** (0.176)	7.298*** (0.242)	7.133*** (0.279)
Province	Yes	Yes	Yes
*t*	2.287	2.076	2.153
DW			1.980
*β*	0.092	0.083	0.086
*F*	5.230**	5.230***	3.481***
*R^2^*	0.008	0.041	0.044

^**^p < 0.05, ^***^p < 0.01; Robust standard error in brackets.

In addition, based on the analysis of the original model, the augmented inverse probability weighting (AIPW) method was adopted to reduce the impact of endogeneity issues and test the results of the benchmark regression. Merge “excellent” and “very good” in the health status into 0, and merge “good,” “not good,” and “poor” into 1. The independent variable becomes a binary variable for analysis. The results are shown in Table 4 and are significant at the 5% statistical level. It can be seen that after applying augmented inverse probability weighting (AIPW) to overcome the sample selection problem, the direction and significance of the results are consistent with the benchmark regression, indicating that the empirical results in this manuscript have good robustness.

**Table 4:** Endogeneity test (augmented inverse probability weighting, AIPW).

Variable	Coefficient	Robust standard error	z	*p*	LLCI	ULCI
ATE	0.360	0.160	2.26	0.024	0.048	0.673

### 4.2 Exploratory analysis of Chinese youth health status on digital health literacy

**Figure 2:**
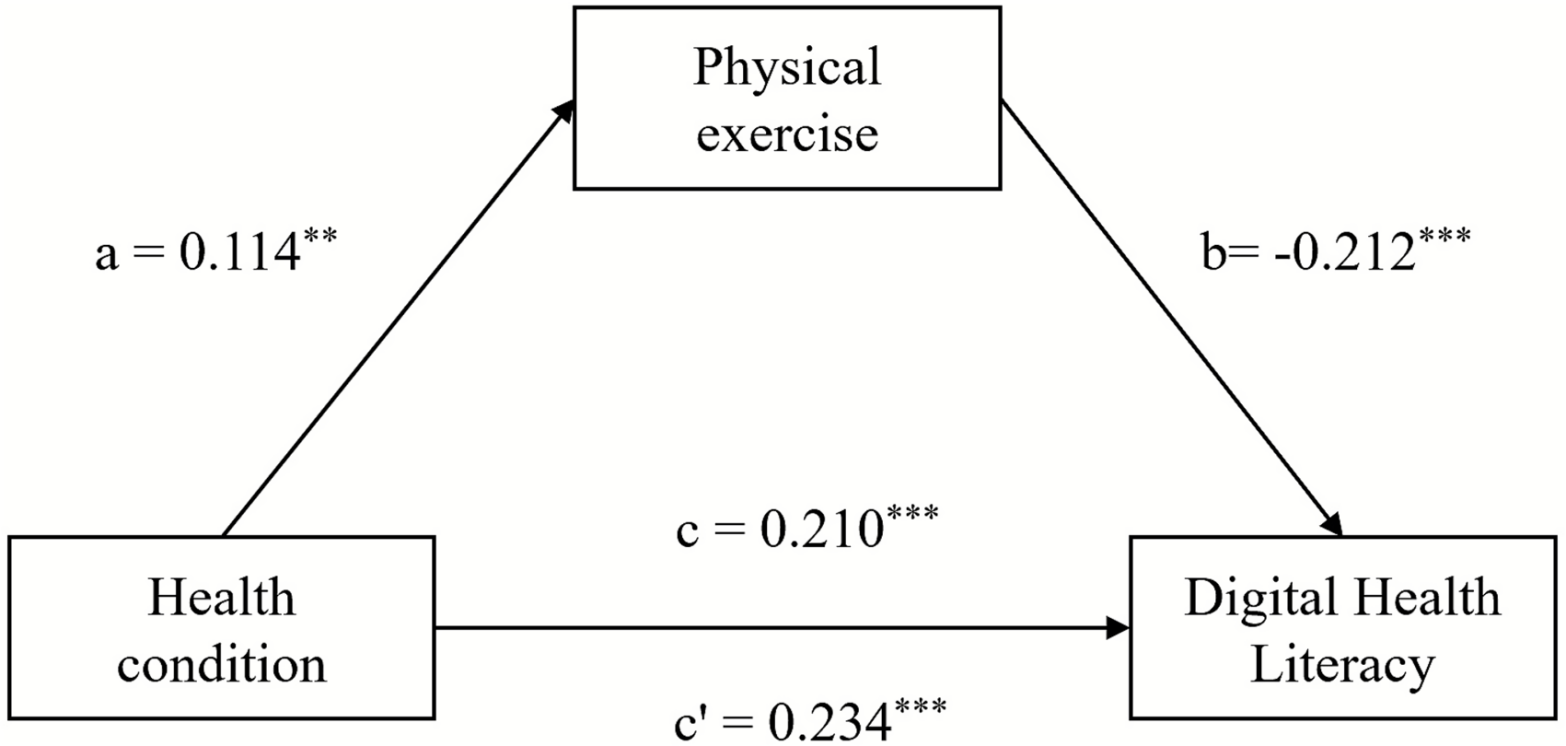
Exploratory analysis of Chinese youth health status on digital health literacy. ^*^p < 0.1, ^**^p < 0.05, ^***^p < 0.01; Robust standard error in brackets.

As mentioned above, health status may affect Chinese youth digital health literacy through physical exercise behavior. In order to explore the intrinsic reasons for the negative correlation between the health status of contemporary Chinese youth and digital health literacy, physical exercise was further added as an intermediate variable to the structural equation model. Tested by using PROCESS’s model 4 in SPSS, and the effect of physical exercise on contemporary Chinese youth health status on digital health literacy was verified and analyzed according to the bootstrap method provided by Hayes. See Figure 2 and Table 5 for specific results.

**Table 5:** Exploratory analysis of Chinese youth health status on digital health literacy.

Variable	Physical exercise			
	Effect	SE	LLCI	ULCI
Total effect	0.210	0.080	0.053	0.367
Direct effect	0.234	0.080	0.077	0.390
Mediating effect	−0.024	0.014	−0.055	−0.002
Control variable	Yes			

The test results are shown in Figure 2 and Table 5. First of all, there is a significant correlation between the health status of Chinese youth and physical exercise, that is the worse health status of youth, the higher frequency of physical exercise (*β* = 0.114, *р* < 0.05). At the same time, the health status of Chinese youth has a significant correlation on digital health literacy (*β* = 0.210, *р* < 0.01), indicating that the total effect is true. Secondly, after adding the variable of physical exercise, the correlation of Chinese youth health status on digital health literacy is still significant (Health status: *β* = 0.234, *р* < 0.01). The direct effect of the health status of Chinese youth on digital health literacy is 0.234, and the indirect effect of physical exercise is −0.024, accounting for 11.4% of the total effect, indicating that physical exercise plays a partially mediating role in the model. The results of this research indicate that physical exercise plays a masking role in the relationship between the Chinese youth health status and digital health literacy. Based on empirical results, the research considers the following. Firstly, from the perspective of time allocation, the process of offline exercise behavior conflicts with online health information acquisition, affecting the improvement of digital health literacy. Secondly, physical exercise is accompanied by the accumulation of offline social capital, while the improvement of digital health literacy usually depends on the formation of online social networks. There is a contradiction between the two in the spatial field.

### 4.3 Heterogeneity of Chinese youth health status on digital health literacy: an analysis based on individual characteristics and socioeconomic status

Combined with the literature analysis on digital health literacy in the previous text, socioeconomic status factors such as education level and income are key variables affecting health outcomes and access to social resources (17). Based on Weber’s “Trinity” social stratification standard and academic practice, socioeconomic status is represented by education and economic status, and individual characteristics are represented by gender and marriage. Explore the different differences in the impact of the health status of contemporary Chinese youth on digital health literacy. For this, subsample regression was conducted by grouping according to gender, marriage, educational level and economic status. Among them, in terms of education, high school and below (including high school) are recorded as medium and low educational qualifications, while those higher education than high school are recorded as high educational qualifications. In terms of economic status, the youth above the average level (including average level) are recorded as high, and those below the average level are recorded as low. Under the condition of controlling other variables, the specific results are detailed in Table 6.

**Table 6:** Heterogeneity analysis based on socioeconomic status.

Variable	Gender		Marriage		Education		Economic situation	
	Male	Female	Unmarried	Married	Medium to low	High	Low	High
Health condition	0.225^*^ (0.126)	0.203^*^ (0.105)	0.291^***^ (0.109)	0.103 (0.120)	0.149 (0.126)	0.295^***^ (0.102)	0.190 (0.155)	0.207^**^ (0.094)
Demographic variables	Yes	Yes	Yes	Yes	Yes	Yes	Yes	Yes
Economic characteristic variable	Yes	Yes	Yes	Yes	Yes	Yes	Yes	Yes
Province characteristic variable	Yes	Yes	Yes	Yes	Yes	Yes	Yes	Yes
Constant	6.253^**^ (0.466)	6.834^***^ (0.424)	6.446^***^ (0.425)	7.125^***^ (0.462)	6.570^***^ (0.512)	7.274^***^ (0.381)	6.647^***^ (0.600)	7.255^***^ (0.321)

^*^p < 0.1, ^**^p < 0.05, ^***^p < 0.01; Robust standard error in brackets.

Firstly, from the perspective of education, there is a significant correlation (*p* < 0.01) between health status and digital health literacy among highly educated youth. From the perspective of economic, there is a significant correlation (*p* < 0.01) between health status and digital health literacy among youth with high economic status. It can be seen from the above, there is significant socioeconomic heterogeneity in the relationship between health status and digital health literacy among contemporary youth. That is the relationship between health status and digital health literacy only shows a significant correlation among highly educated and high-income youth groups. Secondly, from the perspective of marriage, there is a significant correlation between health status and digital health literacy among unmarried youth (*p* < 0.01). In short, there is an unequal impact on the relationship between youth health status and digital health literacy. The higher health status of youth with high economic and social status, the higher digital health literacy. This is consistent with Efrat’s research that the lower the social status of adolescents, the worse digital health literacy (54). This may be due to the fact that this group of youth already has high digital literacy and can actively improve their level of digital health literacy under certain economic conditions, so as to adjust their adverse health conditions in a timely manner (55, 56). And compared to married youth, unmarried youth have more time and energy to pay attention to fluctuations in their own health status, and thus show significant performance in digital health literacy.

## 5 Conclusion and discussion

In the digital age, the digital dependence of the Chinese youth group has become a common phenomenon (57). It is of great significance for youth groups to return to real life, get rid of digital dependence, intervene in health status in advance and strengthen digital health literacy, so as to improve the health status of youth, promote the awakening of youth subjects, and promote national development and national rejuvenation. The main conclusions of this manuscript include the following three aspects:

Firstly, there is a significant negative correlation between Chinese youth health status and digital health literacy. The empirical test of the manuscript found that the weaker health status of youth, the higher their digital health literacy, presenting a “health paradox” phenomenon. This discovery provides a new perspective for health promotion in the digital age and is of great significance. On the one hand, the findings explain the current phenomenon that Chinese youth have high digital literacy, but their health status is still not optimistic. Considering the current social pressure and work pace, as well as the living habits of contemporary Chinese youth, the youth group is tired of running around and busy making a living, knowing that staying up late to work and excessive drinking are adverse behaviors to health, but life is still inevitable due to work needs. Therefore, after the poor health status of Chinese youth or the emergence of diseases, based on the medical demand driven, online consultation, online symptom query and frequent visits to the official websites of relevant health institutions for health information search and other behaviors are carried out at the right time to passively improve digital health literacy to promote health status (58). On the other hand, compared with the existing research, it is found that the Chinese youth group is different from the middle-aged and older adults, and there are significant differences and similarities in the face of health problems. According to the Regulatory Focus Theory, relatively healthy groups tend to maintain the status quo, while relatively unhealthy groups tend to avoid health risks. That is even if improving digital health literacy is beneficial to the health status, most Chinese young groups only pay attention to or improve digital health literacy when health problems occur. After the digital technology enabled medical and health services, the middle-aged and older adults have gradually improved their active health ability and digital health literacy in non-chronic diseases, while the Chinese youth group has stronger digital ability than the middle-aged and older adults, and has not effectively transformed this technical advantage into health promoting behavior, but gradually led to the separation between life and health, showing that the limited health literacy led to the non-ideal health status (59). Specifically, although Chinese youth can skillfully use digital tools to obtain and process health information, their motivation to actively acquire digital health knowledge is weak, and their lifestyles, behavior patterns and health management do not form a synergistic effect with their digital ability. Instead, they rely too much on digital means or ignore the actual implementation of health behavior, resulting in the decline of health level. It can be seen that the Chinese youth as a special group, is different from the student and adolescent groups research by previous scholars, but also differs from the research on the other countries (44, 47, 58). This is highly related to the education system pressure, employment competition pressure, and limited social resource allocation caused by the current social stratification in China, reflecting the unique social characteristics of Chinese youth.

Secondly, Physical exercise has shown a masking effect in the health status and digital health literacy of Chinese youth, presenting a contradictory phenomenon between offline physical exercise and digital health literacy. It is important to note that causal inferences cannot be drawn from cross-sectional data. The current mediation results are exploratory and cannot serve as evidence for causal pathways. Through the empirical test, it has been found that the lower health status of youth, the higher frequency of participation in physical exercise. Meanwhile, physical exercise masks the impact of health status on digital health literacy. In response to the situation, the research considered the following possible factors. Offline physical exercise has crowding out the leisure time of Chinese youth, replacing the cultivation of online digital health literacy and weakened the digital health literacy improvement. Meanwhile, in the process of participating in physical exercise, Chinese youth can experience the multidimensional benefits of physical exercise on health, but it also leads to conflicts between offline exercise behavior and online health information acquisition, which affects the improvement of digital health literacy. Finally, from the perspective of social interaction, physical exercise is accompanied by the accumulation of offline social capital, and the improvement of digital health literacy usually depends on the formation of online social networks.

Thirdly, there is inequality in the relationship between Chinese youth health status and digital health literacy. Socioeconomic status leads to unequal access to information and resources, with a significant correlation observed among youth with high socioeconomic status (high education, high economic status). Referring to digital divide theory, the perception and behavior intervention of Chinese youth with high socioeconomic status on health status is more effective. When the health condition declines, the youth with high socioeconomic status demonstrate stronger adaptability and sustainability due to their better educational background and resource acquisition ability. They can learn and use digital health tools (such as health management applications and online consultation platforms) more quickly, as well as make more effective use of preventive medical services, thereby improving their digital health literacy.

The relationship between Chinese youth health status and digital health literacy is different from previous research. The empirical results show that the better health status of the Chinese youth, the lower their digital health literacy. Over time, this will lead to the phenomenon of “treating only when there is a disease” and the lack of a health concept of “treating before illness.” China is currently in a special stage of comprehensive economic development, and social inequality still exists. At the same time, Chinese youth under social pressure such as employment and work show a sense of powerlessness in health control. Therefore, the Chinese youth tend to ignore health prevention and early intervention, and seek digital media help only when obvious health problems occur. This behavior mode of passive response to health problems is closely related to the high-intensity work rhythm and cruel social pressure of youth. From the perspective of preventive medicine, this health management mode has great risks of chronic diseases and other health problems.

Therefore, it is suggested that the Chinese youth should intervene in advance before the decline of health status. That is “it is best to prevent diseases before they occur.” Firstly, optimize individual health management, improve health status through regular exercise, sufficient sleep and other healthy lifestyles, adhere to regular hospital physical examinations and use health monitoring equipment to control health risk factors, in order to maintain a healthy state. At the same time, actively broaden the level of health knowledge to improve digital health literacy. Secondly, actively participate in community sports activities, health lectures, and digital health experience activities to bridge the digital divide, and enhance digital health literacy through a combination of health data platforms and digital health literacy education. Thirdly, the government to introduce relevant policies to promote the health of the youth. Universities should collaborate with health organizations to develop digital health literacy courses. The governments, communities, and universities as pilot groups, leading the organization of health education activities for the youth population. At the same time, sports competitions and online health incentive activities should be set up for the youth population to enhance their health awareness and digital health literacy through social and cultural changes. So that Chinese youth can avoid health risks, improve health level and realize the sustainable development of a healthy society through the cultivation of digital health literacy and further attention to health status.

## 6 Limitations and prospects

Admittedly, there are limitations to the research. Firstly, although the questionnaire involved in this research has a sufficient sample size and wide coverage, but compared with existing research on digital health literacy, the measurement tools used in this research are limited (60). Due to the limitations of the overall questionnaire, it is not possible to include all dimensions of digital health literacy in empirical analysis, resulting in a relatively narrow measurement range, unable to reflect the complete structure of digital health literacy. Meanwhile, subjective measurements may be influenced by self-perception, and although empirical analysis can be conducted based on self-assessment of health, it is also relatively limited. Therefore, in future research expansion, the research will be made to adopt more comprehensive digital health literacy measurement tools and more objective health indicators (such as diagnostic conditions or medical utilization rates) in order to support better empirical studies. Secondly, the use of data has phased characteristics. The living conditions, work intensity, and employment pressure of youth vary in different periods and backgrounds. At the same time, due to the limitations of measurement tools, there may be measurement biases. Such as poor health levels, leading to immediate demands and frequent searches for health information. Regarding this, the manuscript acknowledges that only relative rigor can be achieved. Finally, due to the limitations of cross-sectional data that cannot support causal inference, the research can only explain some issues through correlation explanations. Meanwhile, the intermediary relationship is explained in the form of exploratory analysis, making the research more rigorous. In the future research, the research will rely on longitudinal (panel) data, natural experiments (exogenous health shocks), or instrumental variable approaches to establish causal chains, in order to provide a more powerful explanation for this mechanism.

## Data Availability

The datasets presented in this study can be found in online repositories. The names of the repository/repositories and accession number(s) can be found in the article/supplementary material.
